# Understanding *Phakopsora pachyrhizi* in soybean: comprehensive insights, threats, and interventions from the Asian perspective

**DOI:** 10.3389/fmicb.2023.1304205

**Published:** 2024-01-11

**Authors:** Md. Motaher Hossain, Farjana Sultana, Laboni Yesmin, Md. Tanbir Rubayet, Hasan M. Abdullah, Shaikh Sharmin Siddique, Md. Abdullahil Baki Bhuiyan, Naoki Yamanaka

**Affiliations:** ^1^Department of Plant Pathology, Bangabandhu Sheikh Mujibur Rahman Agricultural University, Gazipur, Bangladesh; ^2^College of Agricultural Sciences, International University of Business Agriculture and Technology, Dhaka, Bangladesh; ^3^Department of Agroforestry and Environment, Bangabandhu Sheikh Mujibur Rahman Agricultural University, Gazipur, Bangladesh; ^4^Japan International Research Center for Agricultural Sciences (JIRCAS), Tsukuba, Ibaraki, Japan

**Keywords:** *Phakopsora pachyrhizi*, migration, prediction, detection, resistance, fungicides, biological control

## Abstract

Soybean (*Glycine max* L.) is an important crop in Asia, accounting for 17% of global soybean cultivation. However, this crop faces formidable challenges from the devastating foliar disease, Asian Soybean Rust (ASR), caused by *Phakopsora pachyrhizi*, a biotrophic fungus with a broad host range, causing substantial yield losses (10–100%) in Asia. This comprehensive review consolidates knowledge on ASR, encompassing its impact, historical perspectives, genetic diversity, epidemic drivers, early detection, risk assessment, and sustainable management strategies of ASR in the region. ASR has expanded globally from Asia, reaching Africa and Americas, driven by wind-dispersed urediniospores. Genetic diversity studies reveal the complexity of *P. pachyrhizi*, with distinct populations exhibiting varying virulence patterns. Factors affecting ASR epidemics in Asia include host susceptibility, landscape connectivity, climate, and environmental conditions. Understanding the interplay of these factors is essential for early intervention and control of ASR in soybean fields. Effectively managing ASR can exploit the utilization of diverse intervention strategies, encompassing disease forecasting, automated early detection, disease resistance, fungicide application, and biological control. A pivotal aspect of successful, sustainable disease management lies in reducing the ASR pathogen virulence and preventing it from developing fungicide resistance, while the highpoint of effectiveness in disease control is attained through a synergistic approach, integrating various strategies. In summary, this comprehensive review provides insights into multifaceted approaches that contribute to the development of sustainable and economically impactful soybean production in the face of the persistent threat of ASR in Asia.

## 1 Introduction

**Figure 1:**
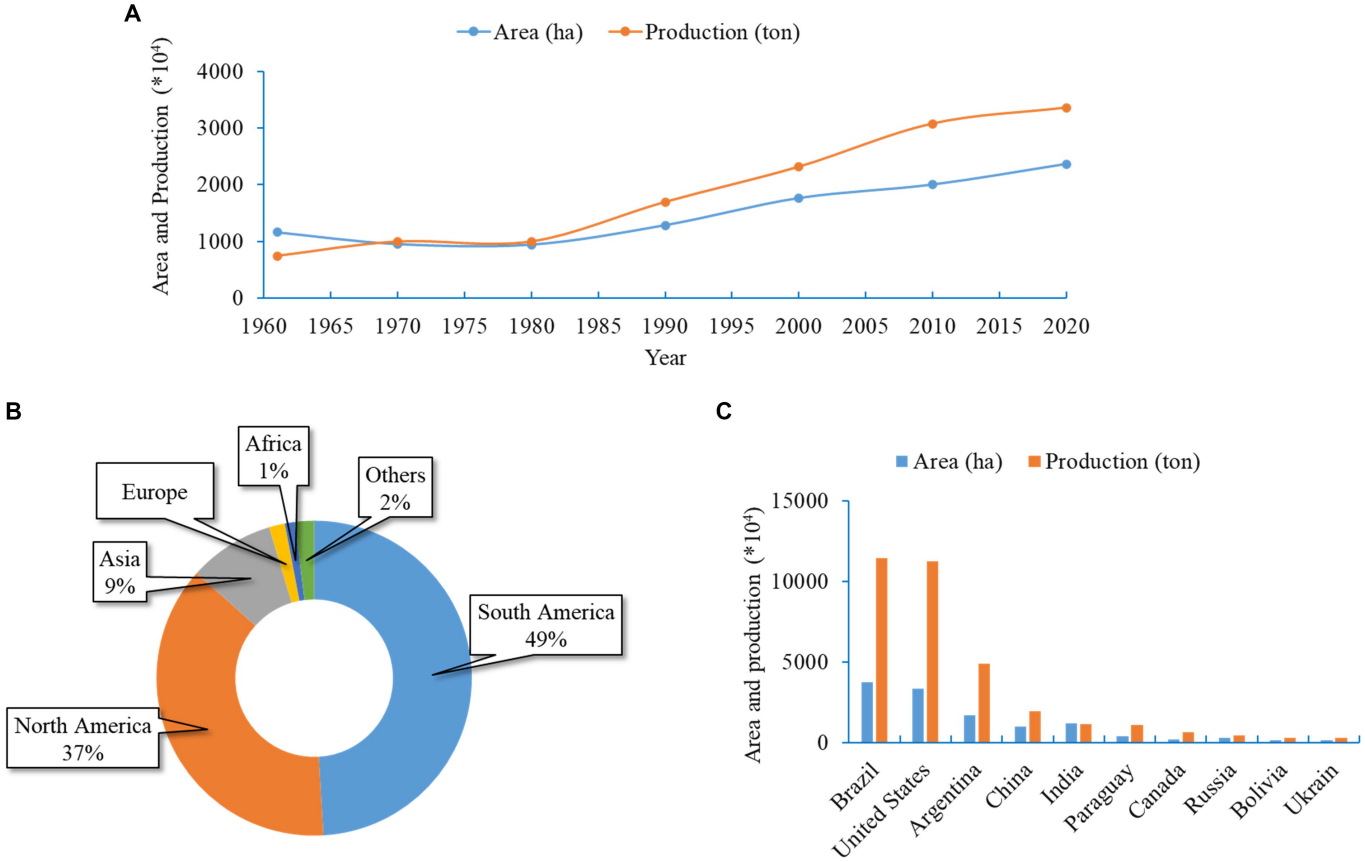
Soybean cultivation area and production in Asia and the world. **(A)** Area and yield of soybean in Asia in different years from 1960 to 2020. **(B)** Global soybean production recorded for individual continents. Average values during 2020 were used. **(C)** Top 10 soybean-producing countries. All data were obtained from FAOSTAT.

**Figure 2:**
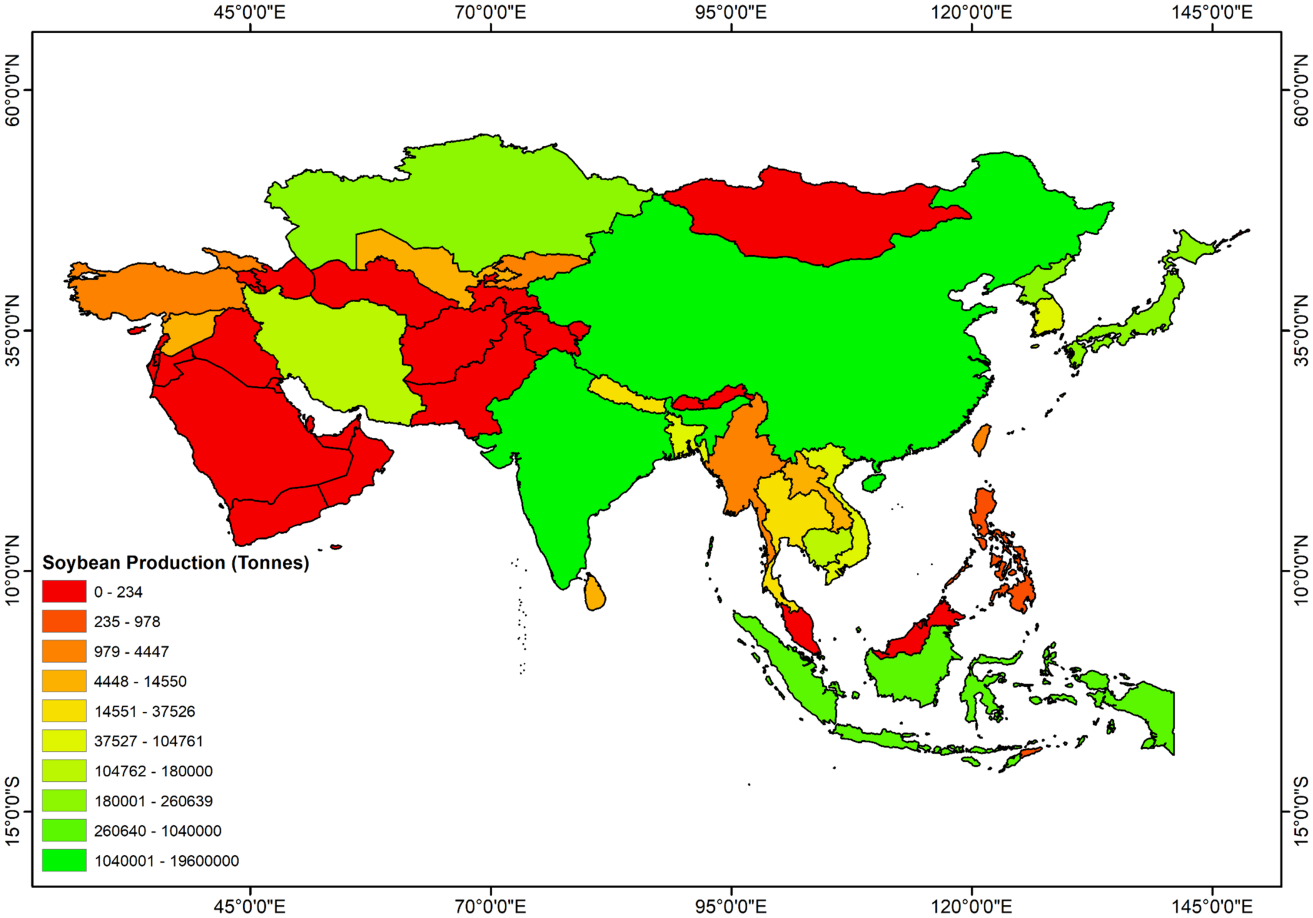
Geographic distribution of soybean production in Asia. All data were obtained from FAOSTAT.

Soybean (*Glycine max* L.), belonging to the family Fabaceae, is one of the most admired and versatile legumes. It is popularly known as the “king of beans” and is grown on every continent worldwide. Currently, soybean is considered the fourth most widely grown crop after the three most important cereal crops; soybean cultivation includes a worldwide planting area of 102.77 million ha with a yearly harvest of 239.36 million tons (FAO, 2020). The crop is grown for food, feed, and other industrial purposes. Due to the wide range of benefits, the global consumption and demand for soybeans are growing at 514 million bushels per year (WAP, 2012). In consequence, the total soybean cultivation area and production grew consistently from the 1960s to the present (Figure 1A). Asian soybean cultivation system represents 17 and 9% of its global cultivation area and yield, respectively (Figure 1B). China and India are two leading global producers of soybeans, holding 4th and 5th positions, respectively (Figure 1C). These two countries report more than 90% of total production in Asia (Figure 2). Indonesia is the third-largest producer in Asia, contributing 3% of the total production, which is followed by Japan (1%) and Myanmar (1%) (Figure 2). In recent decades, soybean production has grown remarkably in India and Bangladesh (FAO, 2022). Although over the past 60 years, the yield per hectare improved substantially (from 6.44 to 14.52 t/ha) across Asia; the estimated average yield is approximately two folds lower than the average global yield (27.91 t/ha) (FAO, 2022). Many biotic and environmental factors may be responsible for the low productivity of soybeans in Asia.

Soybeans are frequently infected by various pathogenic fungi, bacteria, viruses, and nematodes, which significantly affect yield. Asian soybean rust (ASR), caused by the biotrophic fungus *Phakopsora pachyrhizi* Sydow et al. (1906), is a devastating foliar disease posing a substantial challenge to the global soybean sector (Rupe and Sconyers, 2008). This disease has been reported to cause significant yield losses, particularly in poorly managed soybean fields (Sikora et al., 2013). A broad host range is a unique characteristic feature of *P. pachyrhizi* that infects more than 31 species belonging to 17 genera of legumes, ensuring a year-round source of inoculum (Goellner et al., 2010). The capacity of the fungus to incur significant production losses and thrive on a wide variety of hosts reflects the magnitude of the potential threat posed by the ASR pathogen on soybean crops. Therefore, efforts are needed to prevent the fungus from infecting soybean crops, as inaction could result in catastrophe. Active research and serious studies on ASR have been conducted previously, with numerous publications on every aspect of ASR research and the optimization of various management strategies in ASR-affected areas. This review article evaluated the threat and invasiveness of the ASR pathogen in Asia, summarized research on previously reported disease intervention, and suggested a few prospective study areas for future consideration.

## 2 Asian soybean rust, the major constraint of soybean production in Asia

**Figure 3:**
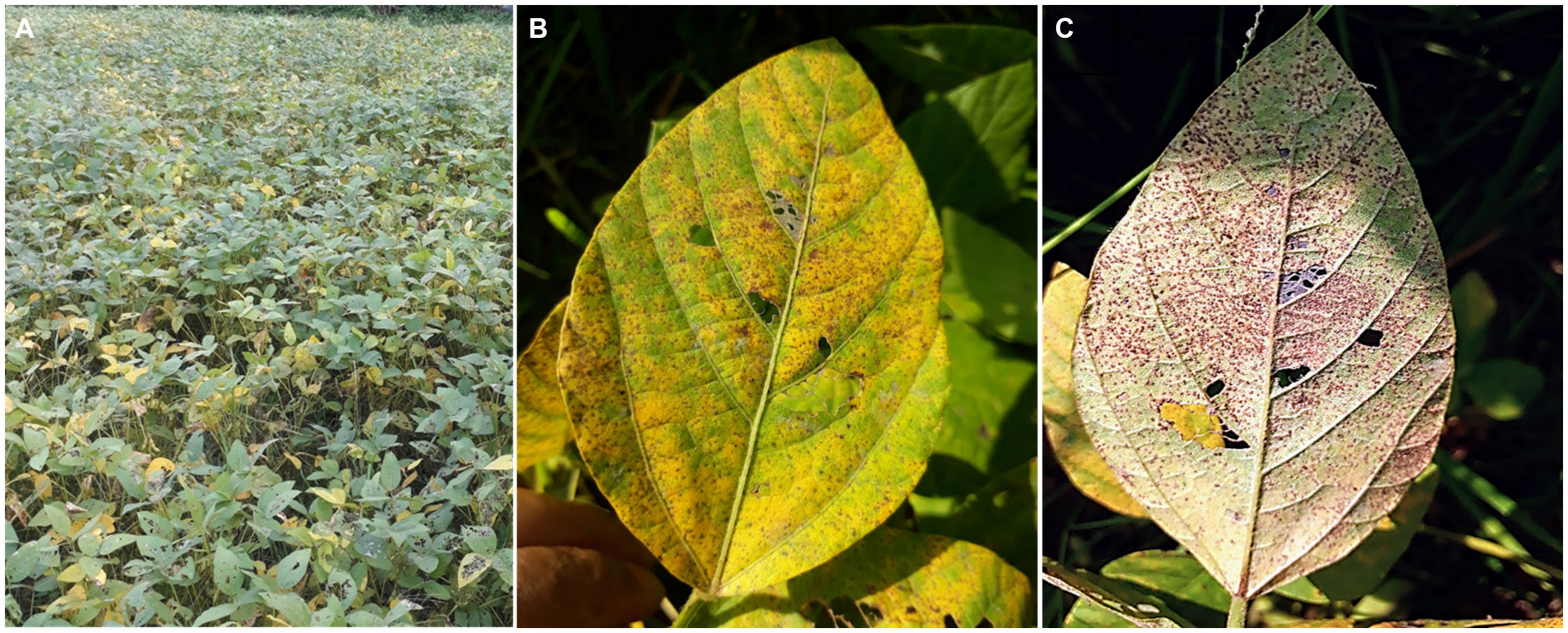
Symptoms of Asian soybean rust (ASR) symptoms in the field. **(A)** A severely ASR-infected field. **(B)** A severely ASR-infected soybean leaf showing yellowing. **(C)** Abundant sporulation of *P. pachyrhizi* from uredinia formed on the lower side of the leaf (the same leaf as **B**).

Due to the continuous expansion of soybean growing area, Asian soybean rust (ASR), caused by *P. pachyrhizi*, may become a more severe problem. Furthermore, global warming will facilitate the spread of infectious pathogens in temperate regions (Chaloner et al., 2021). The weather conditions and naturally existing vegetation of Asia, where ASR originated, favor its perennial survival (Ashok and Naik, 2001; Pivonia and Yang, 2004). The rapid spread and significant yield loss render ASR a dreadful disease in most soybean-growing countries in Asia (Ashok and Naik, 2001). The symptoms of ASR can appear at any developmental stage of plants and are commonly observed on leaves, petioles, and occasionally on the stems (Rupe and Sconyers, 2008). Development of ASR symptoms typically initiates in the lower leaves and gradually progresses in all organs. Although lesions are frequent on the abaxial surface of leaves, they can develop on both leaf surfaces. Small grayish-brown lesions initially appear and eventually turn tan to dark brown (Figure 3). Numerous pustules appear on the dorsal side of the leaves, representing a typical symptom of ASR (Figure 3). Several pustules can coalesce on the abaxial foliar side, and the adaxial foliar surface may become necrotic. As the severity increases, plants senesce prematurely and frequently lose their foliage, leaving petioles attached to the stems. Plants at the flowering stage show the most rapid symptom development and significant defoliation. Premature defoliation or decreased photosynthesis during the flowering to pod-filling stages causes severe yield loss. The earlier the leaves fall, the smaller and fewer grains are formed, leading to a significant decrease in yield.

Different Asian countries have reported varying levels of yield losses, ranging from 10 to 100%. These variations in losses can be attributed to several factors, including the timing of infection, the genotype of the crops being planted, and the prevailing climatic conditions. The disease in China has been documented to cause a decrease in crop yields ranging from 30 to 50%, with the extent of the reduction being influenced by rainfall levels and the severity of infestation (Yu et al., 1994). Before 1977, ASR was not an important disease in India, resulting in minor yield loss. After 1993, ASR often causes 10–90% yield loss (Miles et al., 2003). Taiwan has faced severe economic repercussions since the early 1960s, with estimated yield losses of up to 80% (Chen, 1989; Miles et al., 2003). Similar high yield losses were observed in Thailand (100%), Korea (22.3–68.7%), Indonesia (90%), the Philippines (up to 80%), and Bangladesh, making ASR a significant constraint for soybean cultivation across Asia (Sangawongse et al., 1977; Shin and Tschanz, 1986; Cueva Fe et al., 1994; Hossain and Yamanaka, 2018; Sumartini and Sari, 2022).

## 3 *Phakopsora pachyrhizi*, the Asian soybean rust pathogen

In 1902, Nakanishiki, for the first time, identified ASR in Japan and designated it *Uredo sojae* (Kitani and Inoue, 1960). Since the discovery of ASR in Japan, the name of the causative fungus has been revised several times. In 1903, Yoshinaga sent a rust specimen collected from wild soybeans (*Glycine soja*) grown in Japan to the Hennings of Europe for further diagnosis. Hennings validated the fungus as *Uredo sojae*, a species identical to the previously reported one. In India, Sydow et al. (1906) reported an *Uromyces* belonging to the same Uredoform and found uredospores identical to those of the previously mentioned *Uredo sojae*. They, for the first time, reported teliospores of ASR pathogen in 1906 and recognized the fungus as *P. pachyrhizi* rather than *Uromyces sojae* (Sydow et al., 1906). In 1908, Kawakami and Miura, after their futile attempt to find the teliospores in the ASR occurring in Japan, proposed that *Uromyces sojae* should be used to describe the Japanese isolate of the ASR to distinguish it from *P. pachyrhizi* (Kitani and Inoue, 1960). In 1906 Sydow et al. (1906) described a rust infection in *Pachyrhizus erosus* (L.) Urb. (=*Pachyrhizus angulatus*) grown in Taiwan and referred to the pathogen as *P. pachyrhizi* Syd. & P. Syd, which is the current scientific name. In 1919, Fujikuro observed both uredospores and teliospores on the same leaves infected by soybean rust in Taiwan and agreed with Sydow et al. (1906) in naming this fungus *P. pachyrhizi* (Yang, 1977). Sawada found another rust parasite on soybeans in Japan in 1931 and named it *P. sojae* (Sawada, 1933). However, further studies in 1932 by Hiratsuka demonstrated that this fungus is morphologically identical to *P. pachyrhizi* (Hiratsuka, 1932). In the following year, Hiratsuka concluded that only one fungus was responsible for ASR in soybeans: *P. pachyrhizi*. All other fungi, formerly known as *Uredo sojae*, *Uromyces sojae*, and *Phakopsora sojae* were the same species and were synonymous with *P. pachyrhizi*.

A less aggressive species *Phakopsora meibomiae* (Arthur), has been known to cause ASR in Central and South America since the 1970s (Ono et al., 1992). Both *P. pachyrhizi* Syd. and *P. meibomiae* (Arthur) are morphologically identical, and were considered the same species for many years. Isozyme investigations, for the first time, confirmed that they are two distinct species belonging to *Phakopsora* (Bonde et al., 1988; Ono et al., 1992). Subsequently, sequence comparison of the ITS1 and ITS2 regions of the *P. pachyrhizi* and *P. meibomiae* isolates revealed only 80% similarity (Frederick et al., 2002). The dissimilarity between these two species is also reflected in their geographic distribution, host range, and relative virulence. *P. pachyrhizi* is the causal agent of ASR in Asia that later spreads to Africa, and the Americas, whereas *P. meibomiae* is restricted to the tropical and subtropical climates of the Americas (Schneider et al., 2005; Hartman et al., 2015). Although both ASR pathogens infect a broader range of hosts than that infected by other rust fungi, *P. meibomiae* infects more legume species than *P. pachyrhizi* (Sinclair and Hartman, 1996).

## 4 Global trajectory, the spread of *Phakopsora pachyrhizi* from Asia

After the discovery of *P. pachyrhizi* in Japan and Taiwan, soybean infection by this fungus was first reported in the Philippines (Baker, 1914). Later, ASR was reported on soybeans from Southern China (Tu, 1932) and Korea (Hiratsuka, 1935). However, in Thailand and Cambodia, the disease was identified more than a decade later (Litzenberger et al., 1962; Sangawongse et al., 1977; Sharma and Gupta, 2006). The first Indian report of ASR was erroneously performed on a host identified as *Glycine hispida* (Sydow et al., 1906). Later, Butler (1953) corrected the host as a species of *Mucuna*, and the pathogens on *Mucuna* sp. were *Uromyces mucunae* and *U. sojae* (Sathe, 1972). The first authentic report of ASR in India was recorded in Pantnagar in 1970 (Sarbhoy et al., 1972). The incidence of ASR was first observed in Nepal in 1984 (Joshi, 1985). Since then, global migration has facilitated the spread of *P. pachyrhizi* from Asia to all other soybean-growing regions. Between 1996 and 2003, it spread to Zimbabwe, Uganda, Kenya, Rwanda, Zambia, Nigeria, Mozambique, South Africa, and Cameroon (Akinsanmi et al., 2001; Pretorius et al., 2001; Levy, 2005; Jarvie, 2009). Recently, the disease has been reported in Ghana, the Democratic Republic of Congo, Tanzania, and Malawi (Bandyopadhyay et al., 2007; Ojiambo et al., 2007; Murithi et al., 2015). The spread of ASR into Africa could have been caused by airborne urediniospores. The moist northeast monsoon winds are believed to have dispersed the urediniospores from western India to the East African coast (Levy, 2005). Contrarily, South America was free of ASR until the 2000/2001 season. The disease began to spread and establish in different South American countries between 2001 and 2004 (Rossi, 2003; Stewart et al., 2005; Yorinori et al., 2005). An analysis of nucleotide sequences of the Internal Transcribed Spacer (ITS) region of the ribosomal DNA indicated that the Brazilian rust showed closer ancestral to African than Asian samples (Freire et al., 2008). The transatlantic dispersal of spores by air currents likely played a role in the natural introduction of the ASR into South America.

To investigate the migration of *P. pachyrhizi* urediniospores to the United States of America (Haudenshield and Hartman, 2015). ITS and ADP-Ribosylation Factor (ARF) genes from 59 foreign and archival isolates were sequenced (Zhang et al., 2012), which led to the identification of two groups of global isolates that are endemic to all continents, some of which reside exclusively in Asia. The occurrence of a few *P. pachyrhizi* isolates in Asia, Australia, and the United States, but not in Africa and South America, indicates an alternative migratory pathway for the USA, which did not pass through Africa and South America. Some *P. pachyrhizi* isolates may have arrived from Asia/Australia via Hawaii in the USA. Alternatively, these isolates migrated from Asia to Australia and Hawaii, followed by direct migration to the USA via Australia or Hawaii. The fact that *P. pachyrhizi* with distinctly different virulence was collected at the same time in two regions of Mexico (García-Rodríguez et al., 2022) and that they had characteristics of the respective *P. pachyrhizi* in North and South America supports the possibility that the ASR that occurred in North America was not transmitted from South America. The occurrence of a unique isolate type in the USA raises several possibilities. One possibility is that some *P. pachyrhizi* strains might have been undetected in the USA for quite some time (Zhang et al., 2012). Another possibility is the rapid genetic diversification of previously migrated isolates from Asia, although sexual recombination is lacking to facilitate genetic exchange in *P. pachyrhizi*. On the other hand, however, a report revealed that hyphal anastomosis occurs between *P. pachyrhizi* urediniospore germ tubes, permitting the migration of various nuclei into the complex hyphal network, nuclear fusion, recombination, or chromosome rearrangements (Vittal et al., 2011). Somatic hybridization may result in the reshuffling of genes from generation to generation between isolates of *P. pachyrhizi*, significantly accelerating the genetic diversification of the original isolates.

## 5 Genetic and virulence diversity of *Phakopsora pachyrhizi* in Asia

Diversity in the population and virulence is necessary for pathogen strength; it aids flexibility, adaptation, and the ability to evolve in response to changing environments. As a consequence, it poses a significant threat to the cultivation of soybean varieties with ASR resistance. Several studies have revealed the population structure and genetic diversity of *P. pachyrhizi* at regional levels using molecular data, such as microsatellite markers (Twizeyimana et al., 2011) and DNA sequencing analyses (Freire et al., 2012; Zhang et al., 2012). However, microsatellite markers are difficult to use in studying the population genetics of an obligate biotroph, such as the *P. pachyrhizi* pathogen, because environmental samples are taken directly from field-collected leaves with uredinia, which contain a pool of *P. pachyrhizi* with an unknown level of genetic relatedness (Freire et al., 2008, 2012; Zhang et al., 2012). In some cases, single urediniospore isolates have been used to investigate molecular var*iation* in ASR pathogen through microsatellite marker analyses (Ordoñez and Kolmer, 2007; Vélez-Climent and Dufault, 2007). Under these circumstances, sequence-based approaches involving the internal transcribed spacer (ITS) region and the ADP-ribosylation factor (ARF) appear suitable for unraveling phylogeographic queries regarding environmental samples (Zhang et al., 2012). The phylogenetic analysis based on ITS sequences from a global ASR isolate collection revealed the existence of six distinct clades, whereas ARF sequences indicated only two clades in Asia. Notably, a significant overlap was observed between the phylogenies from ITS and ARF sequences (Zhang et al., 2012), suggesting the need to integrate multi-gene phylogenies to mitigate bias effectively. Moreover, the majority of clads contained isolates of mixed origin (from various countries), indicating that genetic variation at the local level (country) is just as diverse as at the regional level (Asia). The weak genetic structure across large geographic areas was observed not only in Asian populations but also in African and South American populations. In Nigeria, approximately 90% of genetic and virulence variation in *P. pachyrhizi* occurs within soybean fields, with only about 6% variability dispersed across fields (Twizeyimana et al., 2011). In Uruguay, virulence variation within a field exceeds that between fields (Larzábal et al., 2022). In Brazil, samples from three experimental stations exhibited the majority (14 of 16) of ribotypes worldwide (Jorge et al., 2014). The lack of definitive genetic structure across a broad geographic scale indicates efficient mechanisms for long-distance dispersal and significant gene flow between soybean fields.

Studies conducted across Asia, such as in Taiwan, Australia, China, Japan, India, and Bangladesh, have also demonstrated a high level of pathogenic diversity in *P. pachyrhizi* (Table 1). In response to their inoculation onto differential soybean cultivars, varying virulence patterns were observed among *P. pachyrhizi* isolates. For instance, in Taiwan, 50 single-spore *P. pachyrhizi* isolates obtained from Taiwan were used to inoculate five soybean cultivars: PI 462312 (*Rpp3*: Resistance to *Phakopsora pachyrhizi 3*) and TK-5, TN 4, PI 200492 (*Rpp1*), and PI 230971 (*Rpp2*), and were differentiated into three physiological races (Yeh, 1983). In China, four races were identified from seven *P. pachyrhizi* isolates used to inoculate eight soybean entries (Tan and Sun, 1989). In Japan, 18 races were detected after 45 single uredinial *P. pachyrhizi* isolates obtained from cultivated soybeans, wild soybeans, and kudzu (*Pueraria montana*) were used to inoculate a set of 11 different hosts carrying the resistance genes *Rpp1, Rpp2, Rpp3*, and *Rpp4* [74]. Moreover, six races were identified among 26 single-lesion isolates explored in Tsukuba, Japan, while analyzing the pathogen virulence in 13 differential cultivars (Yamaoka et al., 2014). Additionally, the study reported the presence of two or more races of the pathogen in the same soybean cultivar as well as the presence of the same race in both soybean and kudzu. In India, 25 isolates collected from Northern Karnataka from June to October 2010–2012 were differentiated into three pathotypes based on their reactions to 13 hosts (Devaraj et al., 2016). In Bangladesh, 13 *P. pachyrhizi* isolates collected from three soybean-growing regions in 2016 were inoculated onto 12 soybean hosts harboring specific resistance genes soybean-growing regions in 2016 were inoculated onto 12 soybean hosts harboring specific resistance genes (*Rpp1, Rpp2, Rpp3, Rpp4, Rpp5*, and *Rpp6*) and could be segregated into eight pathotypes (Hossain and Yamanaka, 2018). More recently, Schneider et al. (2005) *P. pachyrhizi* isolates collected from five soybean-growing regions in Bangladesh yielded 21 distinct pathotypes (Hossain et al., 2022) suggesting an increasing virulence trend. These findings collectively underscore the dynamic nature of *P. pachyrhizi* virulence in diverse geographical contexts.

**Table 1:** Characteristics associated with of virulence in *Phakopsora pachyrhizi* isolates from different geographic regions in Asia.

Place of isolation	Hosts studied	Number of isolates	Number of Races/pathotypes*	References
Taiwan	Six soybean accessions, and five *Phaseolus* spp.	9	6	Lin (1966)
Australia	PI 200492 (*Rpp1*) and ‘Wills’	Unknown	2	McLean and Byth (1980)
Australia, India, and Taiwan	PI 200492 (*Rpp1*), PI 230970 (*Rpp2*), and PI 462312 (*Rpp3*)	4	4	Bromfield et al. (1980)
Taiwan	PI 200492 (*Rpp1*), PI 230971 (*Rpp2*), PI 462312 (*Rpp3*), TK-5, and TN-4	50	3	Yeh (1983)
Australia	257 accessions of *Glycine canescens* (60), *G. clandestine* (63), *G. tabacina* (100), and *G. tomentella* (47)	8	6	Burdon and Speer (1984)
China	PI 200492 (*Rpp1*), PI 462312 (*Rpp3*), PI 459025 (*Rpp4*), and 5 other accessions	7	4	Tan and Sun (1989)
Japan	PI 200492 (*Rpp1*), PI 230970 (*Rpp2*), PI 230971 (*Rpp2*), PI 462312 (*Rpp3*), Wayne, PI 459024, PI 459025 (*Rpp4*), TK-5, Tainung#4, PI 239871A, and PI 239871B	45	18	Yamaoka et al. (2002)
Taiwan	PI 200492 (*Rpp1*), PI 230970 (*Rpp2*), PI 462312 (*Rpp3*), PI 230971 (*Rpp2*), PI 239871A, PI 239871B, PI 459024 and PI 459025(*Rpp4*), TK-5, TN-4, and Wayne	42	18	Hartman (2011)
Japan	PI 200492 (*Rpp1*), Tainung#4, PI 230970 (*Rpp2*), PI 417125 (*Rpp2*), Ankur (*Rpp3*), PI 459025 (*Rpp4*), Shiranui (*Rpp5*), PI 416764 (*Rpp3*), PI 587880A (*Rpp1*), PI 587886 (*Rpp1*), PI 587905 (*Rpp1-b*), TK-5, and Wayne	26	6	Yamaoka et al. (2014)
India	PI 459024B, PI 459025F, EC-241778, EC-241780, EC-391160, PI 230970 (*Rpp2*), PI 200492 (*Rpp1*), JS-335, PI 230971 (*Rpp2*), PI 459025B, EC-462312, TK-5, and Wayne	25	3	Devaraj et al. (2016)
Bangladesh	PI 200492 (*Rpp1*), PI 587886 (*Rpp1*), PI 230970 (*Rpp2*), PI 462312 (*Rpp3*), PI 416764 (*Rpp3*), PI 459025 (*Rpp4*), PI 200526 (*Rpp5*), PI 567102B (*Rpp6*), PI 587880A (*Rpp1-b*), PI 594767A (*Rpp1-b*), BRS154 (none), and No6-12-1 (*Rpp2, 4, 5*)	13	8	Hossain and Yamanaka (2018)
Bangladesh	PI 200492 (*Rpp1*), PI 587886 (*Rpp1*), PI 230970 (*Rpp2*), PI 462312 (*Rpp3*), PI 416764 (*Rpp3*), PI 459025 (*Rpp4*), PI 200526 (*Rpp5*), PI 567102B (*Rpp6*), PI 587880A (*Rpp1-b*), PI 594767A (*Rpp1-b*), BRS154 (none), and No6-12-1 (*Rpp2, 4, 5*)	34	21	Hossain et al. (2022)

* The number of races/pathotypes here means the number classified by differential varieties in each report.

While soybean differentials, typically composed of soybean cultivars, are commonly used to distinguish pathogenic variation among *P. pachyrhizi* populations, some instances have successfully employed alternative hosts to discern differences in isolate pathogenicity. In the earliest study aiming to dissect *P. pachyrhizi* diversity in Taiwan, nine isolates obtained through single uredospore isolation from rusted soybean leaves were inoculated onto a provisional differential set comprising six soybean varieties and five leguminous plants. Although no significant differences in pathogenicity were observed among the soybean varieties, the isolates were categorized into six pathogenic groups, primarily based on their differential reactions to three leguminous plants: asparagus bean, kidney bean, and short-podded yam bean (Larzábal et al., 2022). In Australia, a pathogenicity test involving eight *P. pachyrhizi* isolates on 257 accessions of various wild *Glycine* species (*G. canescens, G. clandestine, G. tabacina*, and *G. tomentella*) revealed six distinct virulent patterns (Burdon and Speer, 1984). Similarly, an analysis of 10 Kennedia-derived isolates infecting a wild *G. canescens* line and a commercial cultivar of *G. max* identified three distinct patterns of pathogenicity (Burdon and Lenne, 1989). These findings show the potential of differential hosts to unveil the substantial variation within the *P. pachyrhizi* population.

Although the majority of previous reports distinguished isolates based on their virulence profiles in compatible and incompatible interactions with various hosts (qualitative assessment), a quantitative assessment of pathogen aggressiveness could be obtained by assessing host damage or pathogen multiplication (Pariaud et al., 2009). Aggressiveness is quantified based on a variety of quantitative characteristics involved in the host-pathogen interaction, including infection efficiency, latent period, spore production rate, infectious duration, and lesion size (Pariaud et al., 2009). Differences were detected in the aggressiveness of *P. pachyrhizi* isolates collected from Australia, India, Indonesia, and Taiwan (Melching, 1979). Although these isolates required similar times for lesion appearance and secondary uredospore appearance, they differed in terms of the frequency of lesions on leaf area, the time required for lesion appearance, mean lesion area at a given time, the mean number of uredinia per lesion, and mean number and mass of urediniospores produced per lesion on susceptible soybean plants. Based on these criteria, *P. pachyrhizi* isolates vary in their aggressiveness, with Indian isolates being the most aggressive and Australian isolates being the least aggressive (Bromfield et al., 1980). On twenty soybeans analyzed, the reactivity of 10 *P. pachyrhizi* isolates with varying geographic and temporal origins was investigated based on the number of uredinia developed per lesion and the relative sporulation level (Pham et al., 2009). TW 72–1, an isolate from Taiwan, was the most aggressive, whereas IN 73–1 from India was the least aggressive. Similar differences in aggressiveness were reported between U.S. and Nigerian isolates of *P. pachyrhizi* at least in days to lesion appearance, uredinia formation, and uredinia eruption in the susceptible soybean genotype ‘TGx 1485-1D’ (Twizeyimana et al., 2014). Numerous factors can induce large variations in the aggressiveness of *P. pachyrhizi*, including host genotypes, environmental variables, the nature of environment-genotype interactions, and host physiological conditions (Milus et al., 2006; Pariaud et al., 2009; Lannou, 2012). It would be interesting to explore whether the presence of *Rpp* genes influences the level of genetic variation in environmental samples of *P. pachyrhizi*. A study by Jorge et al. (2014) indicated that the presence of *Rpp* (*Rpp1, Rpp2, Rpp3*, or *Rpp4*) genes in hosts did not contribute to a decrease in molecular variation in the pathogen population. This means that using resistant varieties does not result in the selection of highly virulent pathotypes of *P. pachyrhizi.* A possible explanation for this finding is that *P. pachyrhizi* does not reproduce exclusively through asexual reproduction; the pathogen may engage in certain parasexual activities, even though its life cycle in the wild remains poorly characterized. In *P. pachyrhizi*, hyphal anastomosis has been discovered between the germ tubes of the urediniospores (Vittal et al., 2011). When hyphal fusion is followed by heterokaryosis, nuclear fusion, recombination, or chromosome reassortments (Vittal et al., 2011), gene reorganization may occur between generations. The high prevalence of virulence genes in ASR field populations may also explain the absence of a correlation between genetic variation and resistance genes.

## 6 Factors affecting ASR epidemics in Asia

ASR outbreaks have been documented in most soybean-growing regions in Asia since its first report in East Asia, with varying intensities (Echeveste da Rosa, 2015). Factors such as host, pathogen, and environment influence the chance and magnitude of the epidemic. Virulent strains of *P. pachyrhizi* in Japan (Yorinori, 2008; Yamaoka et al., 2014) India (Ramteke et al., 2004; Bhaskar, 2015; Parhe et al., 2017), and Bangladesh (Hossain et al., 2022). can overcome soybean resistance, leading to the onset of epidemics. Landscape connectivity is crucial for the ASR epidemic (Margosian et al., 2009). For instance, regions with concentrated soybean planting as in northern and northeastern China, have a higher probability of ASR epidemics due to frequently available hosts for disease propagation (Li et al., 2010). In contrast, regions with patchy soybean cultivation such as in southern and central China, experience reduced ASR outbreaks due to limited connections between hosts (Li et al., 2010). Mountain ranges protect soybean-growing areas from cold surges, limiting inter-regional spore dispersal. ASR epidemics also depend on the living host and source of the inoculum in overwintering areas (Jurick et al., 2008). Kudzu serves as an ideal overwintering host and a significant initial source of inoculum due to its fast perennial growth and dense canopies. Occasionally, terrain features such as mountain valleys can influence ASR severity in soybean-producing areas by affecting solar radiation, airflow, and average temperatures. In regions such as southern China, where mountain valleys reduce direct sunlight, ASR tends to be more severe (Tan et al., 1996). Mountain ranges protect soybean-growing areas from cold surges (Li et al., 2010) and steep mountains and hills (1,000 m or higher) obstruct low-level airflows, limiting the inter-regional fungal spore dispersal (Pan et al., 2004).

Distinct climatic factors play a significant role in ASR epidemics in different soybean-growing regions in China (Tan et al., 1996). Subtropical regions with abundant summer rain and humid weather during the growing seasons experience frequent and severe ASR pandemics. While summers in the subtropical humid climate are often too hot for *P. pachyrhizi* to infect soybean, the year-round establishment of ASR occurs in overwintering regions during the early growing season. This subtropical region is a significant secondary source of inoculum during the summer months (Giles, 2000). On the other hand, the subtropical wet climate region in southern China is unfavorable for ASR, but serves as a reservoir of urediniospores transmitted continuously to regional ASR epidemics (Tan et al., 1996). The timing and amount of rainfall are key factors affecting the ASR epidemic. Increased rainfall during the growing season promotes disease development and negatively impacts the yield. Areas with higher rainfall, such as western Java in Indonesia, experience more prevalent and severe ASR outbreaks (Bromfield, 1976). In contrast, rain may wash fungal spores away from the air, limiting the spread of *P. pachyrhizi* spores. Ten minutes of rain at a rate of 5 mm per hour can eradicate 50% of *P. pachyrhizi* urediniospores significantly faster than the dry deposition process (Li et al., 2009). During rain, urediniospores tend to clump and stick together, although rain splash facilitates the dislodgement of spores from the uredinia (Del Ponte et al., 2006a).

Temperature also significantly influences ASR during the growing season. Overall, high temperature ranging between 17°C and 27°C is favorable for ASR during the growing season in China, especially in both overwintering (northern) and non-overwintering regions (southern), whereas the disease development is suppressed at a temperature above 30°C (Tan et al., 1996). In Yunnan, ASR develops fairly well except during winter, owing to temperature elevation (the maximum monthly mean temperature ranges between 19°C and 22°C) (Tan et al., 1996). In Australia, a mean daily temperature of less than 15°C slows ASR developments (Casey, 1981). Similarly, Taiwan experiences inhibition or prevention of ASR when mean night temperatures are less than 14°C (Tschanz, 1984). However, the reduced light intensity in cloudy zones favors ASR onset (Li et al., 2010). On the contrary, high daytime temperatures, intense solar radiation, and elevated ultraviolet (UV) radiation are detrimental to *P. pachyrhizi* spore survival (Tan et al., 1994; Isard et al., 2005).

**Figure 4:**
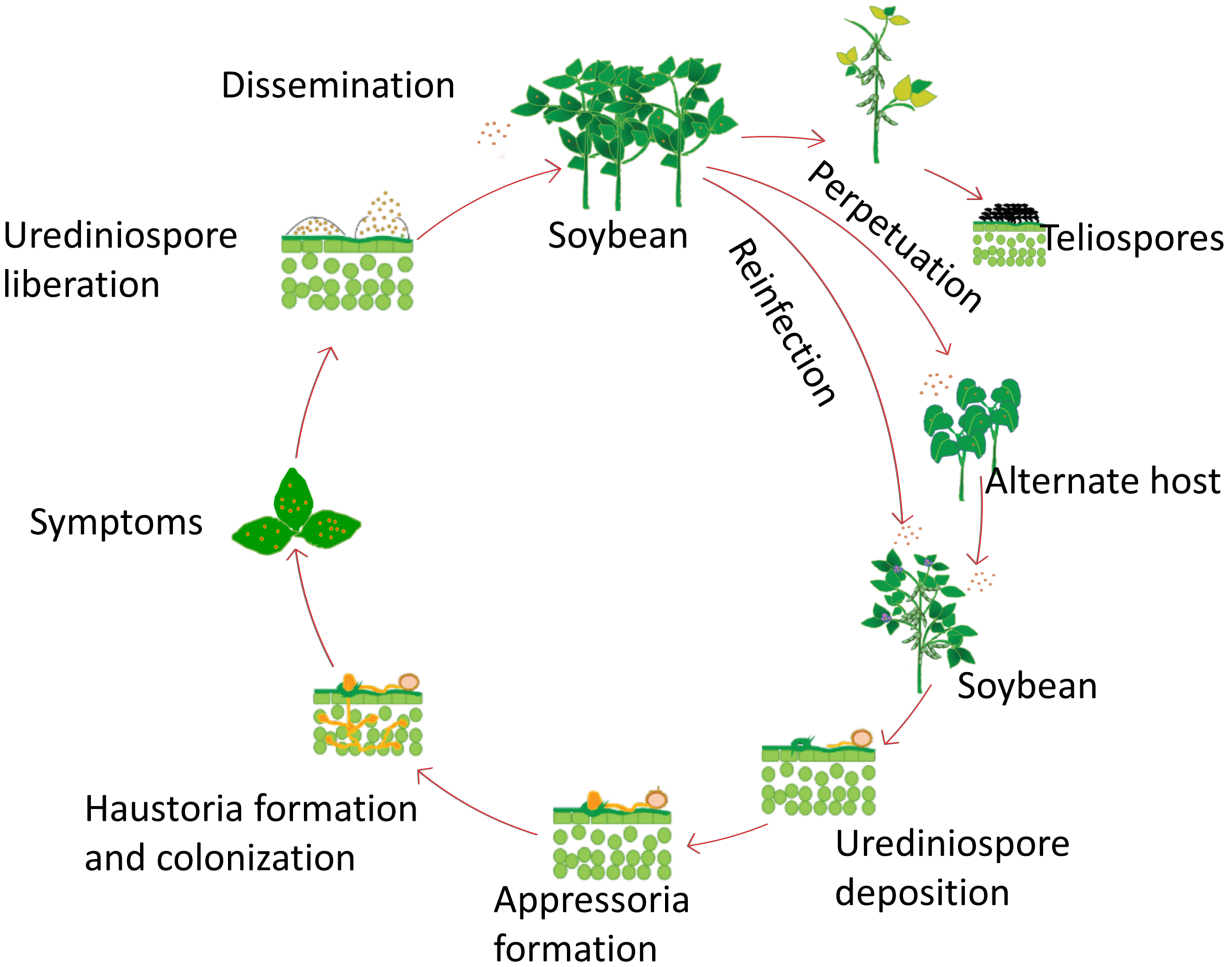
Life cycle of *Phakopsora pachyrhizi*, the Asian soybean rust fungus.

Each component and subcomponent of the ASR cycle is also strongly influenced by various environmental factors. *P. pachyrhizi* has a simple life cycle and produces only urediniospores and teliospores (Mendgen and Hahn, 2002). In this life cycle, the uredinial stage is repeated, producing hundreds of urediniospores in each pustule, which are then released via the ostiole and dispersed by the wind (Figure 4). Infection begins when moist susceptible host surfaces receive urediniospores. Germination of urediniospores requires an optimum temperature of 22–23°C, and free moisture for at least 6 h (Gulya et al., 1997). A longer phase of darkness generally favors the germination and infectivity of urediniospores (Li et al., 2010). Germ tubes always develop from the shady side of spores and extend away from the light source during germ tube development (Koch and Hoppe, 1987). The germ tube differentiates into terminal appressorium without requiring melanin for turgor pressure accumulation in it (Chang et al., 2014). The optimum temperature for appressoria formation generally ranges between 18°C and 23°C, while no appressoria is formed above 30°C (Alves et al., 2006). Unlike other rust fungi, *P. pachyrhizi* directly penetrates host cells instead of entering through stomatal openings. The appressorium accumulates osmolytes, achieving an internal turgor pressure of up to 5–6 MPa, enabling the infection peg to pierce the host epidermis (Chang et al., 2014). The infection leads to intercellular colonization, forming a specialized structure called a haustorium (Micali et al., 2010). Temperatures between 22°C and 26°C promote infection, while no infection occurs at 28°C or higher (Tan et al., 1996). As the intracellular parasitic growth progresses within host cells, the lesions start developing with the development of uredinia, the reproductive pustules. Urediniospores are formed within the uredinia 5–8 days after infection (Goellner et al., 2010). Teliospores are developed on mature old lesions, but cannot germinate in nature (Goellner et al., 2010). Urediniospores can grow at a wide range of temperatures with their optimal range between 20 and 25°C (Gulya et al., 1997). Uredinia passively releases urediniospores; strong winds and air turbulence are the primary factors responsible for spreading *P. pachyrhizi* spores. Strong winds, particularly speedy low-level airflows, transport spores to long distances (Isard et al., 2005). Overwintering of *P. pachyrhizi* on living hosts requires temperatures greater than 4°C, however, urediniospores can survive short freeze/thaw cycles (Jurick et al., 2008).

## 7 An intricate genome of *Phakopsora pachyrhizi*

Publicly available information on the *P. pachyrhizi* genome sequencing project is limited. In 2004, the U.S. Department of Energy Joint Genome Institute (USDoE JGI) Community Sequencing Program initiated a project to sequence the genome of *P. pachyrhizi* isolate Taiwan 72–1 using a fosmid shotgun sequencing approach. However, the initially predicted genome size of 50 Mb proved significantly underestimated. The sequencing project now holds a “permanent draft” status at the JGI.^1^ While the mitochondrial genome sequences of *P. pachyrhizi* and *P. meibomiae* have been released (Stone et al., 2010), information regarding assembly attempts of the nuclear genome remains unpublished. The challenge in nuclear genome sequencing studies for *P. pachyrhizi* lies in the remarkable size and complexity of its genome. According to Posada-Buitrago et al. (2005), the genome size of *P. pachyrhizi* ranges from 300 Mb to 950 Mb, depending on the analytical method. Igor Grigoriev, the head of the JGI fungal program, also supported this claim and suggested that the genome might exceed 850 Mb (Duplessis et al., 2012).

Another effort was known to uncover the genome size of *P. pachyrhizi* isolate Brazil 05–1 involving a k-mer analysis (Loehrer et al., 2014). DNA from urediospores of Brazil 05–1 generated 47 Gb Illumina whole-genome sequencing data (100 bp paired-end reads). Analysis using the JELLYFISH program resulted in an N50 value of 569 bp after assembly and scaffolding with SOAPdenovo. However, it did not provide any insight into gene number or length.

A collaborative initiative comprising 12 governmental and commercial organizations was taken to unravel the *P. pachyrhizi* genome.^2^ The completed genome size was 1.057 Gb, making it one of the largest plant pathogen genomes among fungi. Despite its vast size, *P. pachyrhizi* harbors approximately 20,700 genes, comparable to other rust fungi. Sequencing data revealed that nearly 90% of all sequences consist of non-coding DNA and sequence repeats. The consortium produced a transcriptome map of all fungal structures and infection stages, shedding light on active genome regions throughout the pathogen life cycle.

Gupta et al. (2023) recently published three independent *P. pachyrhizi* genomes. The work conducted by JGI unveiled a 1.25 Gb genome with two haplotypes and a transposable element (TE) content of ~93%, one of the highest documented. Class 1 retrotransposons, constituting 54.0% of the genome, are the most prevalent TE type. The majority of TEs (over 80%) are comprised of two types: long terminal repeats (LTR) and terminal inverted repeats (TIR). Retrotransposons account for a substantial portion, with Gypsy retrotransposons alone representing 43% of the entire genome. The high TE content suggests a strategic approach to increase genetic variation in *P. pachyrhizi*, potentially playing a crucial role in host range adaptation and stress responses.

## 8 Interventions and control of Asian soybean rust in Asia

### 8.1 Prediction and risk assessment

Risk assessment is an epidemiological study that uses non-experimental methods, frequently computer modeling, to forecast the future occurrence of a disease. Many ASR disease models have been developed for the United States of America and South America (Isard et al., 2005; Kim et al., 2006; Pivonia and Yang, 2006; Del Ponte et al., 2006b) however, few are available for Asia. Several risk assessment and prediction models for analyzing the ASR epidemic in Taiwan have been developed (Yang, 1991; Batchelor et al., 1997). SOYRUS T, the first ASR model, was developed at the Asian Vegetable Research and Development Center (AVRDC) to assess ASR epidemics as part of pest risk analysis, and it was validated using experimental data obtained in southern Taiwan during 1979–1981. Considering the dew period and temperature as independent variables, this process-driven computer simulation model simulated a daily increase in disease severity in two soybean varieties (TK5 and G8587) and accurately predicted the infection rate (Yang, 1991). Moreover, the simulator accurately predicted the time of onset and the periods of epidemics in these two soybean cultivars. Subsequently, this model was combined with the SOYGRO soybean model to simulate disease effects on soybean yield and to assess potential crop losses in the United States (Yang et al., 1991). Two additional ASR models were developed based on Taiwanese disease information. A neural network model was developed to predict the ASR severity of soybean cultivar TK5 at AVRDC (Batchelor et al., 1997). Over a two-year period, data from 73 epidemics were combined with 577 observations, and a total of 7 input variables related to ASR progress and weather were created: (1) planting date; (2) days to maturity; (3) the first day of disease appearance; (4) age of plants on the evaluation date; (5) cumulative days of relative humidity greater than 90%; (6) cumulative degree days for rust development; and (7) cumulative degree-days for crop development. Two networks with three or four input variables produced the best results with the highest coefficient of multiple determination (*R^2^*). The three input networks had the lowest mean prediction errors, with a maximum determination coefficient (0.858). Cumulative probability analysis of the Asian soybean rust data (1981) revealed that 54% of cases were within 10% of observed minus predicted deviations and 84% were within 10% of actual disease severity. To simulate ASR severity, a fuzzy logic-based model named FLAIR (fuzzy logic apparent infection rate) was developed and validated using field experimental data based on two soybean cultivars, TK 5 and G 8587, grown in Taiwan (Kim et al., 2005). The FLAIR model assesses the daily apparent infection rate of ASR based on population dynamics and simulates the disease severity. In weekly simulations, the FLAIR model could describe greater than 85% of disease severity variation. Once the baseline values of disease severity were successfully predicted, the FLAIR model could accurately predict disease severity throughout the duration of an epidemic simulation. In China, site-specific calendar-based forecasting models with rainfall as a predictor have been developed to forecast the local severity of ASR (Tan et al., 1994).

To predict infection favorability in Wuhan, an empirical model that considers linear regression analysis of ASR severity based on amount and duration (days) of rainfall during the critical soybean growing season (September and October) was used (Tan et al., 1994). Furthermore, similar predictive models based on local ASR disease and rainfall data were developed in Lishui and Datian (Tan et al., 1994). Integrating climate data into models is critical for identifying distinct climatic conditions suitable for epidemics across different regions. Rainfall patterns appear unsuitable for severe epidemics like those observed in central-western Brazil. However, the disease can develop to light and moderate levels every year if inoculum is available early to mid-season, a condition that may not occur every year because spores must migrate from one to other locations (Del Ponte et al., 2006a,b). Such information is essential for policymakers to determine whether ASR is a major production issue for the region (Yang, 2006).

### 8.2 Automated detection of Asian soybean rust

Timely detection of ASR is crucial for effective management. Visual observation is a common preliminary method, but it often requires specialized skills and subsequent laboratory examinations, which can be costly and time-consuming on large farms. Moreover, the obligate biotrophic nature of the ASR fungus makes it challenging to culture in synthetic media; instead, identification must occur in living plant tissues. To address these challenges, advancements in artificial intelligence (AI), machine learning, big data, and computer vision have significantly improved disease diagnosis. A computer-aided automatic system utilizing image processing technology has been developed to analyze diseased leaf images (da Silva et al., 2022). This system extracts features such as color and texture, enabling quantitative analysis for highly accurate early detection and diagnosis by farmers. Intelligent farming, incorporating image-based approaches with GSM, remote sensing, or other telecommunication technologies, allows remote crop monitoring, aiding in early disease detection and preventing further crop loss (Abdullah et al., 2023).

Recent innovations include leaf-based hyperspectral reflectance for early ASR detection (Furlanetto et al., 2021). Through channel transformation, distribution analysis, and clustering algorithms, ASR-infected leaf images can be classified based on necrosis, chlorosis, and ASR (da Silva et al., 2012). Multispectral image sensors have also been employed, utilizing parameters like the ratio of the infected area, lesion color index, and rust severity index for ASR detection and severity measurement (Cui et al., 2009). In Asia, automatic disease detection and estimation systems have been developed for various foliar diseases of soybeans, including ASR, employing disease severity index, infection per region, and disease level parameters (Shrivastava et al., 2014). However, challenges arise in complex backgrounds, leading to the development of an improved image retrieval-based detection method. This method utilizes various feature extraction techniques and achieves high accuracy in detecting and classifying six soybean leaf diseases, including ASR. With the advent of computer vision technologies, recent efforts have turned to deep learning algorithms for quick and precise ASR detection. In India, a computer vision strategy involves an image processing module and a convolutional neural network (CNN), successfully isolating and recognizing soybean leaf disease symptoms with 98.14% identification accuracy (Shrivastava et al., 2016). Deep learning-based image processing techniques, including smartphone applications, are also gaining traction for disease identification (Verma et al., 2019; Andrianto et al., 2020).

### 8.3 Unmanned aircraft systems for early detection and management of Asian soybean rust

Visual detection limitations have prompted the development of spectroscopic techniques for rapid disease diagnosis (Furlanetto et al., 2021). Studies utilized a portable spectroradiometer and multispectral CDD camera to capture soybean leaf images, varying in rust severity, attempting detection through vegetation indices (Cui et al., 2009; Brodbeck et al., 2017). Unmanned Aerial Systems (UASs) equipped with spectroradiometer and multispectral CDD cameras, emerging as remote sensing platforms, play a significant role in precision agriculture (Liu et al., 2021). UAS offers advantages over larger human-crewed aircraft or satellites due to reduced cost and complexity, rapid data collection, high spatial resolution, and cost-effectiveness (Berni et al., 2009). UAS facilitates quick responses to developing diseases, allowing timely fungicide applications for disease management. It validates large-scale satellite monitoring of ASR and generates prescription maps for site-specific fungicide spraying, reducing yield loss and pesticide use, thus benefiting the environment.

**Figure 5:**
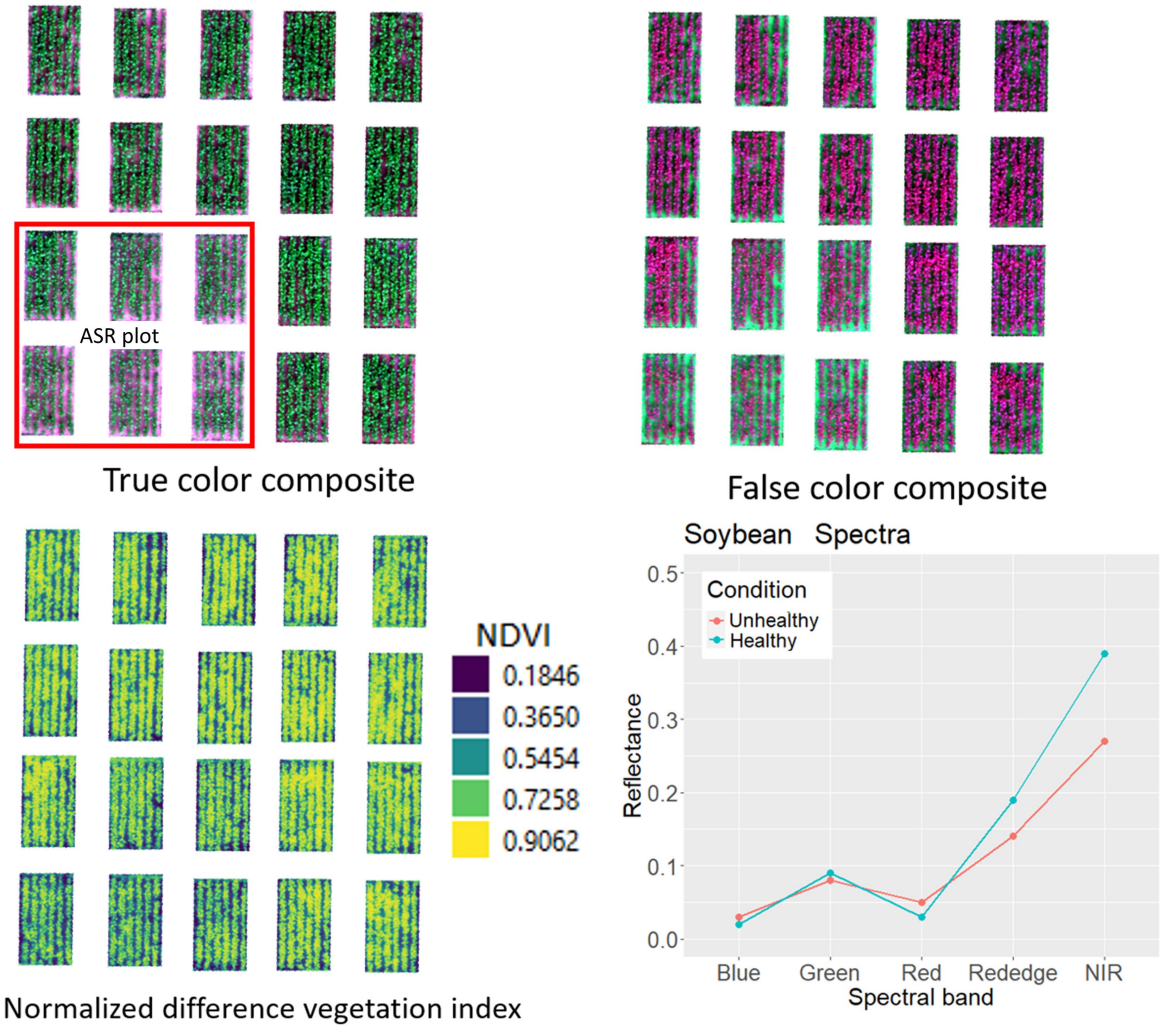
True color and false color multiband imagery of soybean field developed to create crop health index, and spectral curve for healthy and Asian soybean rust diseases canopy at BSMRAU experimental field.

Soybean spectra revealed distinctions in chlorophyll behavior between healthy and ASR-affected plants, reflected in lower NDVI (normalized difference vegetation indices) values for ASR (Figure 5). UAS-based disease scouting introduces a cost-effective strategy for ASR detection in regions where ground-based scouting is impractical, offering significant economic benefits for soybean production in Asia and globally (Sikora and Delaney, 2016). Overall, precise crop management with UAS has the potential to minimize yield losses, enhance input efficiency, and contribute to sustainable and economically impactful soybean production.

### 8.4 Discovery and genetic analysis of Asian soybean rust resistance

**Figure 6:**
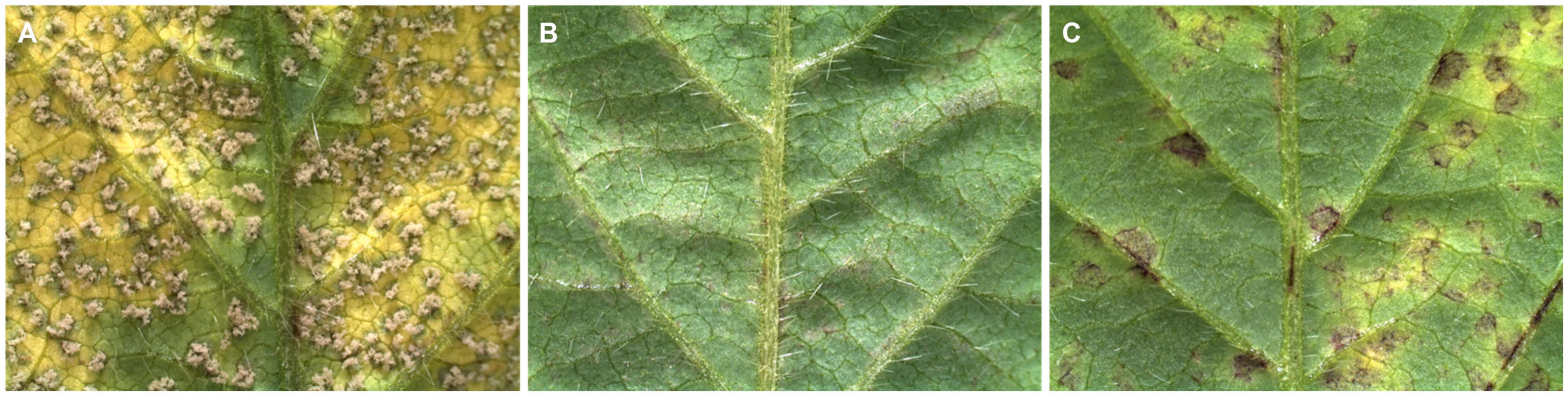
Typical infection phenotypes in different soybean genotypes infected by Asian soybean rust (ASR). **(A)** Susceptible phenotype characterized by abundant *P. pachyrhizi* sporulation on the soybean leaves. **(B)** Immune phenotype characterized by incompatible interaction showing no visible disease symptom on soybean leaves. **(C)** Resistant phenotype characterized by the formation of reddish-brown lesions without fungal sporulation.

Selecting resistant lines against target diseases and developing resistant cultivars are major goals of plant breeding programs in many countries. Plant resistance against *P. pachyrhizi* is often governed by specific resistance genes that interact with avirulent pathogen genes. Based on the absence or presence of the resistance gene, three possible infection phenotypes are exhibited by soybean during interaction with *P. pachyrhizi* susceptible, immune, and resistant phenotypes (Figure 6). Completely susceptible soybean produces tan-colored (TAN) reactions with abundant uredinia and sporulation (Figure 6A). The immune phenotype is presumed to represent an incompatible interaction without any visible disease symptoms and sporulation on the host leaves (Figure 6B). Soybean cultivars that are immune to rust are desirable. However, the absolute immunity to soybean rust is rare in available soybean cultivars. Soybean pyramided line No6-12-1 containing *Rpp2 + Rpp4 + Rpp5* showed an immunity phenotype after inoculation by the BdRP-22 infection (Yamanaka and Hossain, 2019). In contrast, the rust-resistant soybean is generally characterized by the development of reddish-brown lesions (RB) with no or limited pathogenic development or sporulation (Figure 6C). However, some phenotypes are classified as either near-immune resistant phenotypes or intermediate between resistant and susceptible phenotypes, which is attributable to minor effects of genetic factors and the influence of the resistance allele (Yamanaka et al., 2010). The initial soybean screening trials at AVRDC,1977 showed that rust-resistant soybean cultivars are not very common; the screening found no resistant but only two moderately resistant soybean genotypes (G8586 and G8587) (Table 2). Similarly, no highly resistant cultivars were identified in another extensive screening of over 9,000 soybean accessions at AVRDC (Tschanz et al., 1983). Patil and Basavaraja (1997) reported similar observations in India; they assessed numerous soybean genotypes and cultivars under natural epiphytic conditions and discovered no rust-resistant genotypes. Nevertheless, a few genotypes (EC-392530, EC-392538, EC-392539, EC-392541, SL-423, RSC-1, RSC-2, JS-80-21, and PK-1029) exhibited partial resistance against ASR. All 23 soybean lines were susceptible to ASR in a field study conducted under natural epiphytic conditions in Barapani, Meghalaya, India (Baiswar et al., 2012). Two soybean germplasm lines, namely EC 241778 and EC 241780, were identified as promising sources of resistance to ASR after rigorous screening of 982 germplasm lines (Patil et al., 2004). In another study conducted during the Kharif season of 2008 in Dharwad, India, 84 soybean genotypes were tested for ASR resistance in natural epiphytic environments, and these two germplasm lines (EC-241778 and EC-241780) were found as resistant genotypes (Khan and Tyagi, 2013). However, the first rust-resistant cultivar developed in India is DSb 21 (JS 335 × EC 241778), which was developed by the University of Agricultural Sciences, Dharwad, and released in 2015 (Immadi et al., 2022). Apart from demonstrating resistance to ASR, DSb 21 also yields 10–12% more than the popular cultivar JS 335. Conversely, segregating material developed from crosses between the highly yellow mosaic disease (YMD)-resistant cultivar SL 979 and ASR-resistant DSb 21 was screened for resistance to both diseases over 5 years (2016–2020) at two hotspots, Dharwad and Ludhiana. Among the four crosses involved, cross DLSb 1 (SL 979 × DSb 21) exhibited a resistant reaction to both diseases (Basavaraja et al., 2021).

**Table 2:** Effective resistance sources against *Phakopsora pachyrhizi* in different countries in Asia.

Soybean genotype	Conferred gene	Origin of rust isolates or population*	Reference
G8586, and G8587	*Unknown*	Taiwan	AVRDC (1977)
EC-241778 and EC-241780	*Unknown*	India	Patil et al. (2004) and Khan and Tyagi (2013)
DSb 21 (JS 335 × EC 241778)	*Unknown*	India	Immadi et al. (2022)
DLSb 1 (SL 979 × DSb 21)	*Unknown*	India	Basavaraja et al. (2021)
DT 2000 (PI 635999)	*Rpp3*, and *Rpp4*	Vietnam	Pham et al. (2009) and Vuong et al. (2016)

*The host resistance/genes were effective against isolates from these origins.

Between 2006 and 2009, field tests were conducted in Vietnam to evaluate the ASR resistance in selected soybean genotypes (Pham et al., 2009). The DT 2000 (PI 635999) resistance check consistently had the lowest Area Under Disease Progress Curve (AUDPC) units in all five experiments. However, genotypes with known rust-resistance genes (*Rpp1, Rpp2, Rpp3, Rpp4*, and *Rpp5*) did not always exhibit low AUDPC units. The soybean genotype PI 230970 (*Rpp2*) appeared to be the most stable one among all characterized genotypes. Several genotypes with unknown resistance genes, including PI 398998, PI 437323, and PI 549017, showed the lowest AUDPC units in at least one of five experiments (Pham et al., 2010). The ASR resistance of DT 2000 was conditioned by genes/alleles at two genomic regions on Chrs. 6 and 18 that most likely correspond to the *Rpp3* and *Rpp4* loci (Vuong et al., 2016).

Several studies investigated resistance to *P. pachyrhizi* in many well-characterized soybean genotypes in different Asian countries. Most lines showed various reactions at different locations. In India, the soybean genotype PI 200492 (possessing the ASR-resistance gene, *Rpp1*) was immune or resistant during field infection conducted in 2014–2016 (Parhe et al., 2017). However, it was susceptible to the majority of ASR isolates collected between 1993 and 1997 (Yamaoka et al., 2002) but resistant to ASR isolates collected from 2007 to 2009 in Japan (Yamaoka et al., 2014). In field trials conducted in Vietnam between 2005 and 2009 (Pham et al., 2010) and laboratory assays conducted in Bangladesh in 2016, PI 200492 (*Rpp1*) exhibited variable reactions (Hossain and Yamanaka, 2018). Twenty soybean accessions, including PI 200492 (*Rpp1*), PI 200488, PI 416826A, PI 416873B, PI 417120, PI 417503, PI 476905A, PI 567024, PI 567059, PI 605829, PI 605838, PI 605854B, PI 605773, PI 605865B, PI 605885B, PI 605891A, PI 606405, PI 615437, PI 471904 (*Rpp5*), and PI 200487 (*Rpp5*), appeared resistant to the Indian rust isolate IN73-1 (Kendrick et al., 2011). *Rpp1-b* was identified as a new resistant allele of *Rpp1*, and subsequent studies have shown that it shows distinctly different responses to ASR pathogens in different regions. Three *Rpp1/Rpp1-b* genotypes, including PI 587886 (*Rpp1*), PI 587905 (*Rpp1-b*), and PI 594767A (*Rpp1-b*), developed IM or RB lesions after being inoculated with ASR isolates obtained from India, Taiwan, and Thailand in 1973, 1980, and 2001, respectively (Pham et al., 2009; Ray et al., 2009). In Japan, PI 587886 (*Rpp1*), PI 587905 (*Rpp1-b*), PI 594767A (*Rpp1-b*), PI 587880A (*Rpp1-b*), and PI 587886 (*Rpp1*) exhibited resistant reactions, while PI 587886 (*Rpp1*) had a susceptible response to ASR isolates obtained between 2007 and 2009 (Akamatsu et al., 2012; Yamaoka et al., 2014). These genes have also been considered to characterize the pathogenicity of ASR in South and North America (García-Rodríguez et al., 2022). Recent studies considered *Rpp1* and *Rpp1-b* to be distinct genes located in close proximity on chromosome 18 (Hossain et al., 2014; Yamanaka et al., 2016).

PI 230970 (*Rpp2*) induced RB lesions in isolates collected in Taiwan (during1972 and 1980), India (1973), Philippines (1977), and Thailand (2001) (Hartwig and Bromfield, 1983; Bonde et al., 2006; Twizeyimana et al., 2014). PI 230970 (*Rpp2*) was susceptible to more than 50% of the *P. pachyrhizi* isolates obtained from soybean, however, showed resistance against those collected from kudzu in Japan in the 1990s (Yamaoka et al., 2002). PI 230971 (*Rpp2*) produced RB symptoms in 34 of the 50 isolates collected from Taiwan (Yeh, 1983). PI 417125 (*Rpp2*) exhibited resistance against fewer than 50% of the ASR samples collected between 2007 and 2009, whereas PI 462312 (*Rpp3*) was resistant to 30% of *P. pachyrhizi* isolates collected in Japan during the 1990s (Yamaoka et al., 2002, 2014).

As recorded between 2007 and 2009, the *Rpp3* genotypes PI 462312 and PI 416764 were completely resistant to the Japanese *P. pachyrhizi* isolates (Yamaoka et al., 2014); both genotypes were also resistant to all ASR isolates obtained from Bangladesh in 2016 (Hossain and Yamanaka, 2018), however, their efficacy was reduced against ASR isolates obtained during 2018–2019 (Hossain et al., 2022). PI 462312 (*Rpp3*) was resistant to ASR during 2000–2001 (Rahangdale and Raut, 2004; Paul et al., 2015), but became susceptible during 2002–2003 in both glasshouse and natural field conditions in India (Ramteke et al., 2004; Bhaskar, 2015; Parhe et al., 2017).

PI 459025 (*Rpp4*) has been stable in Asia for over two decades (Hartman et al., 2005). With a few exceptions, this genotype was proved to be resistant to *P. pachyrhizi* isolates collected from India, the Philippines, Taiwan, and Thailand (Bromfield, 1984; Bonde et al., 2006; Pham et al., 2009). PI 459025 (*Rpp4*) induced resistance against 70% of isolates collected from Bangladesh in 2016, 2018, and 2019 (Hossain and Yamanaka, 2018; Hossain et al., 2022). In Japan, PI 459025 (*Rpp4*) was reported to be resistant to 44 of the 45 *P. pachyrhizi* isolates collected between 1993 and 1997 (Yamaoka et al., 2002), and 21 of the 26 isolates collected between 2007 and 2009 (Yamaoka et al., 2014).

PI 200526, which has long been known as a cultivar possessing *Rpp5*, has recently been shown also to possess *Rpp3* (Yamanaka et al., 2023). PI 200526 showed resistance to all 26 isolates belonging to six races identified in Japan during 2007–2009 (Yamaoka et al., 2014), and all 13 isolates belonging to eight pathotypes obtained from Bangladesh in 2016 (Hossain and Yamanaka, 2018). The frequency of R reactions in PI 200526 (*Rpp5*) was reduced to 78.6 and 35.0% in 2018 and 2019, respectively, against isolates obtained from Bangladesh (Hossain et al., 2022). These results indicate that changes in resistance reactions in soybean plants harboring *Rpp* genes are occasionally observed in some soybean-growing areas.

The Indonesian cultivar PI 567102 B carrying *Rpp6* showed resistance against Indian, Taiwanese, and Japanese *P. pachyrhizi* isolates (Paul et al., 2015), and was primarily found to be susceptible to ASR reported from Bangladesh in 2016, 2018, and 2019 (Devaraj et al., 2016; Hossain and Yamanaka, 2018). These results show that each genotype selectively showed resistance to specific *P. pachyrhizi* isolates found in Asia.

### 8.5 Effectiveness of gene pyramiding for broader and strong ASR resistance

Gene pyramiding is widely regarded as an optimal breeding strategy that develops strong, ASR-resistant soybean varieties. Multigenic interactions between *Rpp* genes can lead to increased resistance in a diverse array of *P. pachyrhizi* isolates (Yamanaka et al., 2013; Panho et al., 2022). To date, only a few studies have been conducted in Asia targeting the development and evaluation of *Rpp*-pyramided soybean lines showing resistance against ASR isolates. The pyramiding of eight combinations of *Rpp2, Rpp4*, and *Rpp5* genes was evaluated for ASR-resistance against the Japanese isolate T1-2 (Yamanaka et al., 2013). The *Rpp*-pyramided line that carried *Rpp2 + Rpp4 + Rpp5* showed a significantly lower frequency of lesions with uredinia (%LU), the number of uredinia per lesion (NoU), and sporulation level (SL) compared to their ancestral accessions An76-1 (*Rpp2 + Rpp4*), PI 230970 (*Rpp2*), PI 459025 (*Rpp4*), and BRS184, except for PI 200487 (*Rpp5*) that was resistant to T1-2 infection and later found to possess another resistance gene, *Rpp3* (Table 3; Yamanaka et al., 2023).

**Table 3:** Reaction phenotypes of *Rpp*-pyramided lines against Bangladeshi and Japanese *Phakopsora pachyrhizi* isolates.

Pyramided genes and its source*	Pyramided lines	Efficacy against Bangladeshi rust isolates	Efficacy against Japanese rust isolate	References
PI 200487 (*Rpp5*) PI 230970 (*Rpp2*) PI 459025 (*Rpp4*)	No2-4F_3_-3 No2-4F_3_-22 No2-4F_3_-25 No2-4F_3_-28 No2-4F_3_-33 No6-12F_3_-1 No6-12F_3_-2 No6-12F_3_-7	NT**	R, HR, or I phenotypes***	Yamanaka et al. (2013)
Hyuuga (*Rpp3*) PI 459025 (*Rpp4*)	Oy15-3	R, HR, or I phenotypes	NT	Yamanaka and Hossain (2019)
PI 459025 (*Rpp4*) PI 200487 (*Rpp5*)	No6-12-B	R, HR, or I phenotypes	R	Yamanaka and Hossain (2019) and Yamanaka et al. (2015)
PI 459025 (*Rpp4*) Hyuuga (*Rpp3*)	Oy49-4	R, HR, or I phenotypes	R	Yamanaka and Hossain (2019) and Yamanaka et al. (2015)
PI 230970 (*Rpp2*) PI 459025 (*Rpp4*) PI 200487 (*Rpp5*)	No6-12-1	R, HR, or I phenotypes	R	Yamanaka and Hossain (2019) and Yamanaka et al. (2015)
PI 459025 (*Rpp4*) Hyuuga (*Rpp3*) PI 200487 (*Rpp5*)	Py6-1-17	R, HR, or I phenotypes	NT	Yamanaka and Hossain (2019)
PI 200492 (*Rpp1*) PI 230970 (*Rpp2*) PI 459025 (*Rpp4*)	Mo42-1	R, HR, or I phenotypes	NS****	Yamanaka and Hossain (2019) and Yamanaka et al. (2015)
PI 200492 (*Rpp1*) PI 230970 (*Rpp2*) Hyuuga (*Rpp3*)	Py4-4-5	R, HR, or I phenotypes	NT	Yamanaka and Hossain (2019)
PI 230970 (*Rpp2*) Hyuuga (*Rpp3*) PI 200487 (*Rpp5*)	Py8-1-15-6	R, HR, or I phenotypes	NT	Yamanaka and Hossain (2019)

*PI 200487 and Hyuuga, which are known to have multiple genes, only the genes listed in parentheses are used in the pyramided lines; **NT-Not tested; ***R-Resistant, HR-Highly Resistant, I-Immune phenotype; ****NS-Not significantly different from source parents.

Moreover, the *Rpp*-pyramided lines required a significantly longer (1.7 day more) incubation period (IP) for ASR lesion development than that recorded in the case of their ancestors (Yamanaka et al., 2013). These results indicate that *Rpp* gene pyramiding provides higher ASR resistance and delays lesion development. Later, resistance to Japanese *P. pachyrhizi* isolate T1-2 was comparatively analyzed in four combinations of double and three combinations of triple *Rpp* genes (*Rpp1, Rpp2, Rpp4*, and *Rpp5*) (Yamanaka et al., 2015). Three *Rpp*-pyramided lines, such as No6-12-B (*Rpp4 + Rpp5*), Oy49-4 (*Rpp2 + Rpp3 + Rpp4*), and No6-12-1 (*Rpp2 + Rpp4 + Rpp5*), showed the highest resistance to the tested isolate (Japanese rust isolates T1-2) compared to resistance observed in their parental sources, including PI 230970 (*Rpp2*), Hyuuga (*Rpp3*), PI 459025 (*Rpp4*), and PI 200487 (*Rpp5*) (Yamanaka et al., 2015). Recently, Yamanaka and Hossain (2019) evaluated the resistance of six *Rpp*-pyramided lines with different combinations of genes, along with seven previously evaluated lines (Lemos et al., 2011; Yamanaka et al., 2011, 2015) against 13 *P. pachyrhizi* isolates from Bangladesh; the di- and tri-*Rpp* lines had higher ASR resistance than that in their mono-*Rpp* parental counterparts, conferring resistance phenotype in 95.6 and 100.0% of infections, respectively, compared with 72.3% for the resistance source varieties. Moreover, these *Rpp*-pyramided lines lacked sporulation and uredinia formation in 35.2 and 73.1% of infection cases, respectively, compared to that recorded in only 13.8% in resistance source development. Later, resistance to Japanese *P. pachyrhizi* isolate T1-2 was comparatively analyzed in four combinations of double and three combinations of triple *Rpp* genes (*Rpp1, Rpp2, Rpp4*, and *Rpp5*) (Yamanaka et al., 2015). Three *Rpp*-pyramided lines, such as No6-12-B (*Rpp4 + Rpp5*), Oy49-4 (*Rpp2 + Rpp3 + Rpp4*), and No6-12-1 (*Rpp2 + Rpp4 + Rpp5*), showed the highest resistance to the tested isolate (Japanese rust isolates T1-2) compared to resistance observed in their parental sources, including PI 230970 (*Rpp2*), Hyuuga (*Rpp3*), PI 459025 (*Rpp4*), and PI 200487 (*Rpp5*) (Yamanaka et al., 2015). Recently, Yamanaka and Hossain (2019) evaluated the resistance of six *Rpp*-pyramided lines with different combinations of genes, along with seven previously evaluated lines (Lemos et al., 2011; Yamanaka et al., 2011, 2015) against 13 *P. pachyrhizi* isolates from Bangladesh; the di- and tri-*Rpp* lines had higher ASR resistance than that in their mono-*Rpp* parental counterparts, conferring resistance phenotype in 95.6 and 100.0% of infections, respectively, compared with 72.3% for the resistance source varieties. Moreover, these *Rpp*-pyramided lines lacked sporulation and uredinia formation in 35.2 and 73.1% of infection cases, respectively, compared to that recorded in only 13.8% in resistance source varieties carrying single *Rpp* genes. Among the 13 lines, two di-*Rpp* (Oy15-3 [*Rpp3 + Rpp4*] and No6-12-B [*Rpp4 + Rpp5*]), and six tri-*Rpp* lines (Oy49-4 [*Rpp2 + Rpp3 + Rpp4*], No6-12-1 [*Rpp2 + Rpp4 + Rpp5*], Py6-1-17 [*Rpp3 + Rpp4 + Rpp5*], Mo42-1 [*Rpp1 + Rpp2 + Rpp4*], Py4-4-5 [*Rpp1 + Rpp2 + Rpp3*], and Py8-1-15-6 [*Rpp2 + Rpp3 + Rpp5*]) showed the highest levels of resistance to *P. pachyrhizi* isolates obtained from Bangladesh (Yamanaka and Hossain, 2019; Table 2). According to a recent study, *Rpp*-pyramided line No6–12–1, carrying *Rpp2, Rpp4*, and *Rpp5*, could confer robust resistance to Bangladeshi ASR samples that are virulent to all *Rpp1–Rpp6* genes (Hossain et al., 2022). The pyramided lines No6-12-B (*Rpp4 + Rpp5*), Oy49-4 (*Rpp2 + Rpp3 + Rpp4*), and No6-12-1 (*Rpp2 + Rpp4 + Rpp5*) also conferred more resistance than the resistance of the source cultivar against the Brazilian isolate BRP-2.6 (Yamanaka et al., 2015), suggesting that *Rpp*-pyramided lines can provide higher resistance to *P. pachyrhizi* isolates of broader geographic origin. These studies indicate that the correct combination of *Rpp* genes in pyramided lines influences the pyramiding effect. Combining appropriate *Rpp* genes in a single genotype can induce improved and broad-spectrum ASR resistance. Pyramiding of resistance genes has already been used to develop ASR-resistant soybean varieties. New ASR-resistant varieties, JFNC 1 and JFNC 2, were recently released in Paraguay (Kato and Soares, 2021). JFNC 1 and JFNC 2 were developed by introducing three resistance genes (*Rpp2, Rpp4*, and *Rpp5*) into the susceptible original Aurora and YG203 varieties, respectively.^3^ These new varieties showed remarkably improved ASR resistance to a diverse range of *P. pachyrhizi* populations in the field showing satisfactory yield under fungicide-free conditions as the original susceptible cultivars yielded, which was grown using fungicides (Kato and Soares, 2021). Subsequently, a new cultivar., ‘Doncella INTA-JIRCAS,’ developed using the same breeding strategy in Argentina, has been released in 2022.^4^

While using resistant varieties is the most preferred strategy for managing ASR, breeding for ASR-resistant cultivars poses several major challenges. The soybean rust pathogen is known for its high genetic variability (Hossain et al., 2022). Identifying novel sources of resistance and the optimal *Rpp* gene combinations against the highly variable ASR populations is the most challenging task for achieving effective resistance deployment. Continuous monitoring and updating of resistance genes are necessary to keep pace with the evolving pathogen. Some ASR resistance is quantitative in nature and controlled by multiple genes with small individual effects (Hossain et al., 2014). This makes it challenging to develop cultivars with stable resistance, as the accumulation of minor genes may not provide long-term protection against evolving pathogen populations. It can also be challenging for any breeding programs to strike a balance between achieving high levels of ASR resistance and maintaining desirable agronomic traits, such as yield, maturity, and seed quality. Careful selection of soybean cultivars that can combine strong resistance with optimal performance in various growth conditions is needed. Another significant obstacle is the time-consuming process of introgressing *Rpp* genes into resistant cultivars. Incorporating modern breeding technologies, such as molecular markers and genomic selection, can accelerate the breeding process. However, the adoption and integration of these technologies into ASR breeding programs may face challenges related to cost, infrastructure, and expertise. Addressing these challenges requires a multidisciplinary approach, involving plant breeders, pathologists, molecular biologists, and other experts to develop soybean cultivars with stable and effective resistance against ASR.

### 8.6 Effective fungicides for managing Asian soybean rust in Asia

An ideal method for controlling ASR is to grow cultivars with high levels of resistance or tolerance. However, such cultivars are scarce in areas where ASR is a serious problem. Therefore, fungicide application is necessary to manage diseases and achieve higher yields. Choosing the most suitable fungicide is critical for effective disease management. In the past, most of the work on fungicidal control of ASR was conducted in Asia (Miles et al., 2003). In particular, AVRDC comprehensively tested fungicides against ASR, but did not continue extensive investigation on soybeans. Mancozeb was one of the earliest and most effective fungicides controlling ASR (Yang and Vakili, 1978). When multiple mancozeb doses were sprayed on soybean cv. KH #3, Shih-Shih, and Wakajima plots, yield protection was recorded to range from 23 to 50% (Yang, 1977). Other fungicides, such as Bayleton 25 WP, Bavistin C-65, benomyl, and chlorothalonil, were as effective as mancozeb for managing ASR in AVRDC field trials (AVRDC, 1977, 1987, 1992). Most of these fungicides are protectant fungicides requiring application prior to infection and remaining on the leaf surface. However, protectant fungicides are less effective in cases of high severity (Miles et al., 2007). Thus, their frequent applications (7–10 days) can reduce the rate of ASR development under heavy inoculum pressure.

Fungicide trials conducted in India have identified several triazole compounds and mixes (Patil and Anahosur, 1998) to be effective against ASR. Among these, flusilazole + carbendazim, difenoconazole, and triadimenol were the most effective ones. In Karnataka, India, a field trial under irrigated conditions demonstrated the efficacy of three triazole fungicides, including hexaconazole (Contaf), propiconazole (Tilt), and triadimefon (Bayleton), against ASR. Three applications of 0.1% hexaconazole spray at 1-month intervals significantly reduced the disease severity and produced higher seed yield, seed weight, less AUDPC, and the highest benefit–cost ratio. Two sprays of 0.1% hexaconazole at 15-day intervals starting from the disease initiation have significantly reduced the ASR by 75–85% and increased the grain yield by 85–135% (Jones et al., 1989). International trials for fungicide efficacy showed that triazole and triazole + strobilurin sprays were the most effective in lowering ASR and improving yields in locations with higher disease severity (Miles et al., 2007). These fungicides are absorbed into leaf tissue and distributed translaminarly, making them the most effective when used prior to infection. The heterocyclic compound triazole inhibits lanosterol 14α-demethylase, an enzyme essential for the biosynthesis of ergosterol, a key fungal cell membrane component, thus inhibiting fungal growth (Cycoń et al., 2006). In contrast, strobilurin fungicides manage ASR by inhibiting spore germination and preventing germ tube development.

### 8.7 Biological control of Asian soybean rust

While fungicides are commonly applied for controlling ASR, their continuous use leads to environmental pollution, higher costs, and fungicide resistance in *P. pachyrhizi* strains (Dorighello et al., 2015; de Paula et al., 2021). To address these problems, Alternative management approaches are being explored globally with a growing interest in biological control agents due to concerns about pesticide residues and sustainability (van Lenteren et al., 2017). Studies from Brazil and the USA have shown the effectiveness of biological agents like *Bacillus subtilis*, *B. pumilus*, and coffee oils against (Ward et al., 2012). ASR in both greenhouse and field conditions (Ward et al., 2012; Gauthier et al., 2014; Dorighello et al., 2015; Twizeyimana and Hartman, 2019). Field application combining *B. subtilis* and fungicides decreased disease severity and increased yield compared to controls (Dorighello et al., 2015). Notably, a biopesticide based on *B. subtilis* QST 713 demonstrated high efficacy *in vitro* and *in vivo*, alone or in combination with synthetic fungicides (Twizeyimana and Hartman, 2019). *Simplicillium lanosoniveum*, a mycophilic fungus, showed promise by inhibiting urediniospore germination and reducing pustule numbers on soybean leaves (Ward et al., 2012; Gauthier et al., 2014). Romeo SC^®^, an elicitor based on *Saccharomyces cerevisiae* cell wall, and Bio-imune^®^, a product formulated with *B. subtilis* BV02, were introduced in Brazil to induce resistance to *P. pachyrhizi*, with the former considered environmentally low-risk (Ward et al., 2012; Mapa, 2022). Overall, these biological control agents offer potential alternatives to fungicides for managing ASR, provide, ng effective disease control with reduced environmental impact and potential economic benefits.

In Asia, the biological control of ASR has been less explored. Isolates of *Verticillium psalliotae* and *V. lecanii* from Taiwan and Thailand and Trichothecium roseum from India have demonstrated efficacy against *P. pachyrhizi* (Saksirirat and Hoppe, 1990; Saksirirat et al., 1996; Kumar and Jha, 2002). A study in India investigated various fungi for resistance against ASR using a detached leaf assay, revealing the mycoparasitic behavior of *T. roseum* on rust pathogens after the 8th day (Pankaj et al., 2014). In field evaluations in Dharwad, India, seed treatment with *T. harzianum* and cow urine showed promising results, with minimal rust disease index and optimal seed yield (1.72 t/ha) (Jahagirdar, 2014). However, the lowest disease and maximum seed yield were obtained using chemical fungicide Hexaconazole (Jahagirdar, 2014). Essential oils from *Hyptis marrubioides*, *Aloysia gratissima*, *Cordia verbenacea*, *Corymbia citriodora*, *Cymbopogon nardus*, *Azadirachta indica*, and *Thymus vulgaris* exhibited inhibitory effects on *P. pachyrhizi* urediniospore germination and greenhouse control of ASR (Medice et al., 2007; da Silva et al., 2012). Treatment with essential oils altered fungal structures such as urediniospores, appressoria, germ tubes, and paraphyses (da Silva et al., 2014), indicating their potential as antifungal agents. While these examples highlight the efficacy of biocontrol agents in ASR management, the cost–benefit ratio and feasibility of implementing biocontrol on a field scale need assessment to determine their agronomic value within integrated pest management (IPM) strategies (Langenbach et al., 2016).

Merely applying individual biocontrol microbes may not achieve sustained effects in suppressing ASR in the field. Therefore, current efforts are focused on enhancing and facilitating plant-associated microbial communities, known as microbiomes, for disease control (Ali and Imran, 2022). Microbiomes encompass a diverse array of microbes with various functions related to plant growth and health. The collaborative effects of these community members lead to more robust, consistent, and enduring impacts, effectively countering the negative effects of plant diseases. Consequently, there is considerable optimism surrounding biocontrol strategies that involve developing a plant-optimized microbial community. The attraction of specific microbiomes in the rhizosphere is linked to signaling molecules, hormones (Carvalhais et al., 2013), and specific root exudates (Carvalhais et al., 2015) released by plants to meet their needs. In turn, the microbiota or microbiome influences plant health and growth through their dynamic activities (Vorholt, 2012). Microbiomes represent one of the most critical factors that influence the success or failure of a defense strategy in controlling plant pathogens (Morra et al., 2010; De Corato, 2020). Despite this, there is a lack of information on manipulating the plant microbiome for the biocontrol of ASR. Therefore, conducting a thorough investigation on soil microbiota and how its manipulation can improve suppression of ASR becomes imperative. A comprehensive understanding of plant communication systems with the microbiome and the specific attraction of microbiomes to soybeans has the potential to provide valuable knowledge for enhancing crop yields and developing ASR-resilient plants without chemical interventions.

## 9 Conclusion and future perspectives

ASR is among the most hazardous diseases affecting soybean crops, causing total output losses during epidemic conditions. Reasonable prediction and diagnosis of *P. pachyrhizi* infections are crucial for early intervention to prevent disease spread across the field, leading to reduced yield loss. Although disease management heavily relies on fungicidal chemicals, their effectiveness may diminish as resistance emerges among fungal populations. Practically all main fungicide groups are affected by the emergence of resistance in fungal populations. Concurrently, the reduction in the durability and effectiveness of resistant genes is primarily attributed to the substantial selective pressure exerted by the ongoing cultivation of the same cultivar. The resulting evolved strains have the potential to render host resistance ineffective. In such a situation, gene pyramiding can be considered a primary approach, as the *P. pachyrhizi* population comprises a complex of races with multiple virulence factors. However, the success of this strategy depends on the availability of the source germplasm, resources, facilities, the time required to release the cultivar., and *P. pachyrhizi* races. Emphasis should be placed on pre-breeding, considering wild relatives of soybeans to broaden the range of resistance sources. Various other strategies, from conventional to advanced techniques, can be suggested, including multi-line formation, combining ability studies, and molecular techniques, such as marker-assisted selection and genetic transformation. Recently, nanomaterials have gained significance owing to their adjustable physical, chemical, and biological properties, along with technological breakthroughs. These new-generation chemical compounds need to be explored for their efficacy against ASR. Alternatively, resistance in crop plants could be improved at genetic levels using modern biotechnological solutions, such as a multiplex CRISPR/Cas9 system in which a cassette of sgRNA is constructed. This can simultaneously edit or target the most conserved sections of host genomes. To effectively utilize these new technologies, the function of the responsible genes and the target DNA sequences need to be identified. Furthermore, developing technologies that can be applied to a wide range of soybean varieties and prevent the introduction of unsuitable phenotypes caused by off-target mutations is necessary.

## Author contributions

MH: Funding acquisition, Project administration, Supervision, Writing – original draft, Writing – review & editing. FS: Writing – original draft, Writing – review & editing. LY: Writing – original draft, Writing – review & editing. MR: Writing – original draft, Writing – review & editing. HA: Writing – original draft, Writing – review & editing. SS: Writing – original draft, Writing – review & editing. MB: Writing – original draft, Writing – review & editing. NY: Funding acquisition, Project administration, Supervision, Writing – original draft, Writing – review & editing.

## Footnotes

^1^ https://genome.jgi.doe.gov/portal/

^2^ https://mycocosm.jgi.doe.gov/Phapa1

^3^ https://www.jircas.go.jp/en/reports/2019/r20190822

^4^ https://www.jircas.go.jp/en/publication/research_results/2022_c01
